# Buckwheat (*Fagopyrum esculentum* M.) Sprout Treated with Methyl Jasmonate (MeJA) Improved Anti-Adipogenic Activity Associated with the Oxidative Stress System in 3T3-L1 Adipocytes

**DOI:** 10.3390/ijms14011428

**Published:** 2013-01-11

**Authors:** Young-Jun Lee, Kui-Jin Kim, Kee-Jai Park, Bo-Ra Yoon, Jeong-Ho Lim, Ok-Hwan Lee

**Affiliations:** 1 Department of Food Science and Biotechnology, Kangwon National University, Chuncheon 200-701, Korea; E-Mails: hslyj02@gmail.com (Y.J.-L.); yoonboll@naver.com (B.-R.Y.); 2 Department of Cancer and Cell Biology, University of Cincinnati College of Medicine, Cincinnati, OH 45267, USA; E-Mail: kimkj@ucmail.uc.edu; 3 Korea Food Research Institute, Gyeonggi 463-746, Korea; E-Mail: jake@kfri.re.kr

**Keywords:** buckwheat (*Fagopyrum esculentum* M.) sprouts, methyl jasmonate (MeJA), anti-adipogenic activity, 3T3-L1, ROS production, obesity

## Abstract

Buckwheat sprouts contain various bioactive compounds including rutin which have a number of biological activities. We have previously shown that buckwheat sprouts (TBWE) treated with methyl jasmonate (MeJA) significantly increased the amount of phenolics and the antioxidant activity. The aim of this study was to demonstrate the effect of TBWE on anti-adipogenesis and pro-oxidant enzyme in 3T3-L1 adipocytes. We also evaluated the anti-oxidative activity of TBWE in adipocytes by using the nitroblue tetrazolium assay. Our data showed that TBWE markedly inhibited adipocyte differentiation and ROS production in 3T3-L1 cells compared with control groups. Moreover, TBWE has strongly shown the inhibition of adipogenic transcription factor as well as pro-oxidant enzymes. Together, we demonstrate that the MeJA treatment significantly increased the amount of phenolic compound, resulting in the suppression of adipogenesis and ROS production in the 3T3-L1 cells. These findings indicate that TBWE has the potential for anti-adipogenesis activity with anti-oxidative properties.

## 1. Introduction

Obesity has emerged as a metabolic disorder associated with cardiovascular disease and type 2 diabetes mellitus [1,2]. Obesity is characterized by an increased storage of triacylglycerol in adipose tissues. Cellular and molecular studies on the development of obesity have shown that increases in the number and size of adipocytes can be triggered by dietary factors [3]. The development of adipose tissue requires the coordinated activation of several pathways initiated by up-regulation of the initiation of the nuclear peroxisome proliferator-activated receptor gamma (PPARγ), CCAAR/enhancer-binding protein α(C/EBPα) and adipocyte protein 2 (aP2) also plays a crucial role in the differentiation of the pre-adipocytes into adipocytes [4].

The production of reactive oxygen species (ROS) has recently been implicated as an important contributor to the pathogenesis of obesity-associated insulin resistance, and the accumulation of body fat in obese individuals leads to the elevation of systemic oxidative stress [5]. When oxidative stress occurs, oxidation of important macromolecules including proteins, lipids, carbohydrates, and DNA ensues. A number of studies have demonstrated potent inhibition of oxidative stress with certain antioxidants and suppression of adipocyte developments with associated nitric oxide synthase and NADPH oxidase (NOX) protection under *in vitro* conditions [6,7]. Thus, using bioactive compounds to control the expression of adipocyte makers, ROS related genes and NOX would be important in the prevention and intervention of adipogenesis.

A number of approaches have shown that bioactive compound extracts from natural products have a therapeutic potential for obesity [8–10]. Rutin (quercetin-3-*O*-rutinoside) has been shown to decrease oxygen radical capacity and adipogenic differentiation markers in adipocyte differentiation [11–13]. Rutin is found in many types of fruits and buckwheat. Buckwheat and buckwheat sprouts have been used as a food ingredient and as a medicinal material worldwide [14,15]. In recent years, buckwheat sprouts have been under the spotlight in the international market because they contain high levels of phytochemicals, including rutin and proteins, which have a number of biopharmaceutical activities [16,17].

We have previously shown that methyl ester (methyl jasmonate, MeJA) induces production of secondary metabolites during sprouting buckwheat and may result in an increase in biopharmaceutical potential of buckwheat sprouts [18]. MeJA plays a central role in regulating the biosynthesis of many secondary metabolites, including flavonoids, and also are signaling molecules in the case of environmental stresses. The main objective of this study was to determine the anti-adipogenesis potential of MeJA treated buckwheat sprout (TBWE). We performed molecular bioassays in 3T3-L1 adipocytes and found that TBWE derivatives suppressed the expression of PPARγ, C/EBPα and aP2, which are major markers for adipocyte differentiation. We measured the glucose-6-phosphate dehydrogenase (G6PDH) and NOX4 transcriptional activities of TBWE during adipocyte differentiation. We also evaluated the oxidative-stress activity of TBWE in adipocytes with a nitroblue tetrazolium (NBT) assay as well as the gene expression of glutathione peroxidase (GPx) and copper and zinc superoxide dismutase (Cu/Zn-SOD).

## 2. Results and Discussion

### 2.1. Total Phenolic Compounds and Antioxidant Capacity of Buckwheat Sprouts Extracts

The phenolic compounds are an important group of secondary metabolites, which are synthesized by plants and physical processing as a result of enhance to antioxidant activity and resistant stress [19,20]. Total phenolic compounds and antioxidant capacity of natural products is an important for its bioactivities. Therefore, we were interested in the evaluation of nutritional value expressed as content of total phenolic compounds and antioxidant capacity of NBWE and TBWE.

Total phenolic compound and antioxidant activities of the NBWT and TBWE are shown in Table 1. The TBWE have not only increased in total phenolic compound (22.9 ± 0.6 mg/g), but also altered the rutin content (2.99 ± 0.11 mg/g) compared to NBWE (14.4 ± 0.4 mg/g and 2.09 ± 0.27 mg/g, respectively).

The antioxidant potentials of the NBWE and TBWE were estimated from their ability to reduce TPRZ-Fe (III) complex to TPTZ-Fe (II). The FRAP values for the NBTE were significantly lower than the TBWE (42.9%). The ORAC values measured in terms of trolox equivalent (TE) between the NBTW and TBWE. Although, it was significantly increased the hydrophilic ORAC value of TBWE (103.3 ± 19.2 μM TE/g) compared to the NBWE (60.6 ± 3.8 μM TE/g). However, there were no significant differences of total antioxidant activities (hydrophilic+lipophilic) contents between the NBWE and TBWE groups.

The previous report showed that rutin inhibited the adipocyte differentiation due to inhibition of adipogenic markers [21]. Table 1 showed TBWE have strongly increased rutin content as well as the phenolic compound compared to NBWE. These results indicate TBWE has a strong potential effect of anti-adipogenic activity compared to rutin. Therefore, we investigated the effect of TBWE on cell viability, before we tested anti-adipogenic activity of TBWE, by using the MTT assay.

### 2.2. Cell Viability of Buckwheat Sprouts Extracts in 3T3-L1 Preadipocytes

To examine cytotoxicity of TBWE, NBWE and rutin, these extracts at a concentration of 50 μg/mL were treated on 3T3-L1 adipocytes for 24 h.

**Figure 1:**
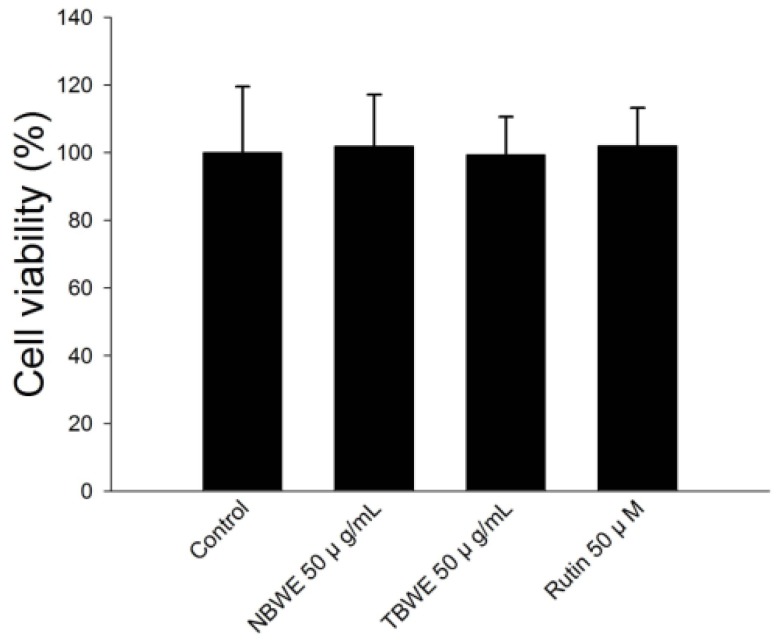
The effect of buckwheat sprout extracts treated with MeJA (TBWE) on the cell viability in 3T3-L1 preadipocytes. The cell viability was determined using the XTT assay. All values are represented as the mean ± standard deviation of five independent experiments.

As shown in Figure 1, TBWE and NBTW-treated cells displayed no significant inhibitory effects on cell viability during the 24-h incubation for any of the groups tested. Here, we show that these extracts had no cytotoxicity along the entire concentration range up to 50 μg/mL, and the concentration which was used was 50 μg/mL for the experiments below.

### 2.3. TBWE Suppresses the Expression of Adipocyte Markers

Terminal differentiation involves the coordinated regulation of several pathways of gene expression by activating the transcription factors that initiate C/EBPα and PPARγ. These two transcription factors are known to promote a late-stage adipocyte marker, aP2 [22,23]. We evaluate the expression of the early-stage differentiation genes, C/EBPα and PPARγ, and the late-stage adipogenic marker, aP2, which are crucial for adipocyte development [10,24].

**Figure 2:**
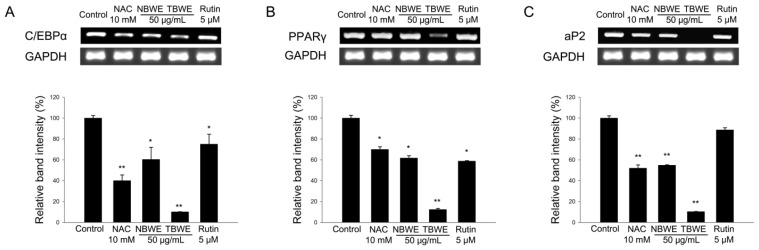
Buckwheat sprout extracts treated with MeJA (TBWE), which suppresses the (**A**) C/EBPα, (**B**) PPARγ and (**C**) aP2 transcription factors during adipocyte differentiation. The results are given as the mean ± standard deviation of three replicates. Within each treatment group, the means without a common letter differ significantly (* *p* < 0.05; ** *p* < 0.01).

As shown in Figure 2, the expression of C/EBPα and PPARγ mRNA was induced in the fully differentiated control, whereas this induction was abrogated by the 50 μg/mL TBWE where it showed a dramatic decrease compared to the results of the 10 mM NAC, 50 μg/mL NBWE and 5 μM rutin treated groups. The expression of C/EBPα in cells treated with 50 μg/mL NBWE, 50 μg/mL TBWE and 5 μM rutin decreased by approximately 39.6%, 89.8%, and 24.9%, respectively; PPARγ was also inhibited by approximately 30.1%, 87.6%, and 41.1%, respectively, compared with the control group (Figure 2A,B). Moreover, the expression of aP2 also significantly decreased by 54.8%, 89.6%, and 11.2% after treatment with 50 μg/mL NBWE, 50 μg/mL TBWE and 5 μM rutin, respectively (Figure 2C). In addition, there were consistent decreases in the levels of C/EBPα, PPAR γ and aP2 in the 10 mM NAC and 5 μM rutin treated groups compared with the control group. The levels of these three adipocyte markers were strongly decreased in the 5 μg/mL TBWE treated group by a greater amount than in both of the 10 mM NAC and 5 μM rutin treated groups.

### 2.4. The Effect of TBWE on Lipid Accumulation and ROS Production in 3T3-L1 Cells

The effect of TBWE on lipid accumulation was analyzed during the differentiation of 3T3-L1 cells using Oil Red O staining. Oil Red O staining is an assay that can detect intracellular lipid accumulation. Induced lipid accumulation by activating C/EBPα, PPARγ and aP2 has been shown during adipogenesis in 3T3-L1 [10].

**Figure 3:**
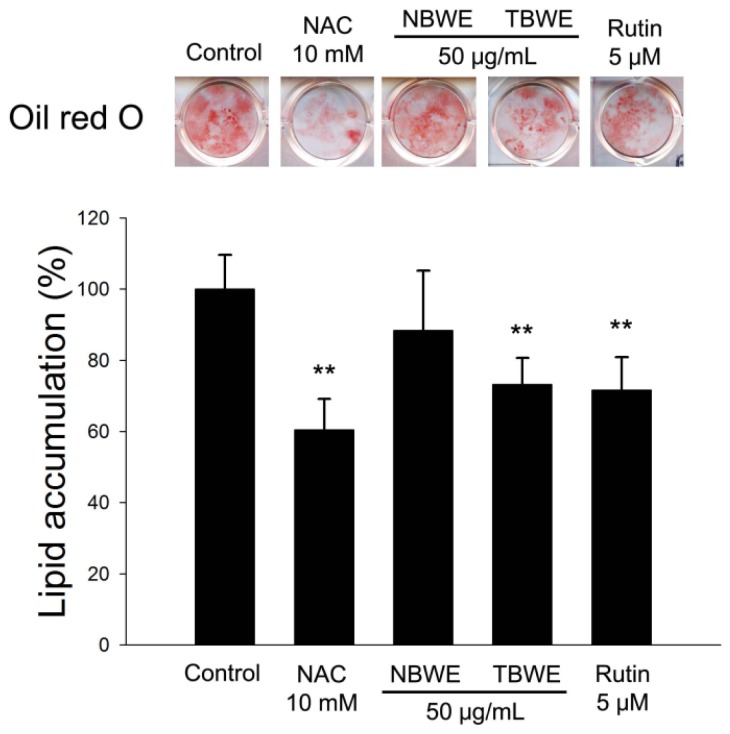
Buckwheat sprout extracts treated with MeJA (TBWE), which inhibits lipid accumulation during the differentiation of 3T3-L1 adipocytes. Lipid accumulation determined by absorbance at 570 nm. Each value given is the mean ± standard deviation of five individual plates and is representative of the results from at least five independent experiments (* *p* < 0.05; ** *p* < 0.01).

The control group had lipid accumulation strongly induced in differentiated 3T3-L1, whereas the TBWE-treated cells showed a significantly reduced accumulation of intracellular lipids compared with the control group, as shown in Figure 3.

**Figure 4:**
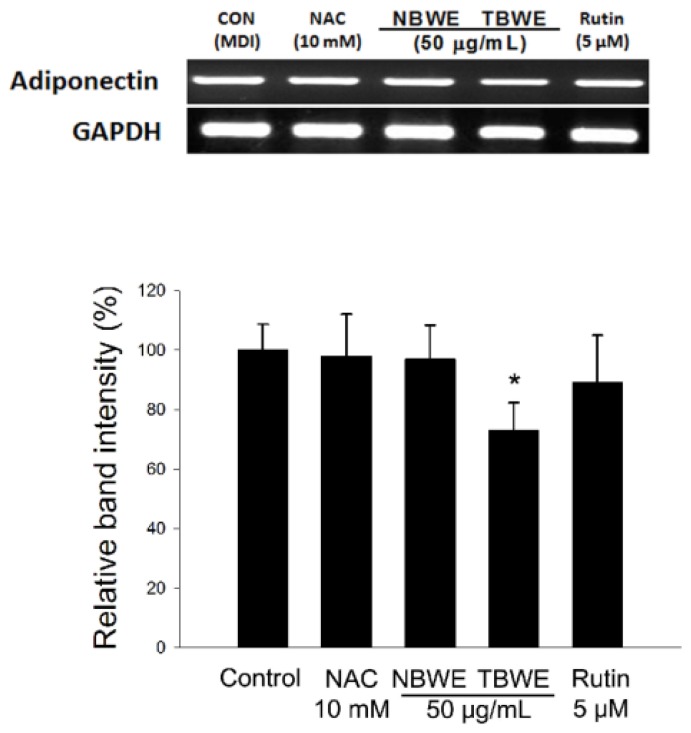
Effect of Buckwheat sprout extracts treated with MeJA (TBWE) on adiponectin mRNA expression in 3T3-L1 cells. The results are given as the mean ± standard deviation of three replicates. Within each treatment group, the means without a common letter differ significantly (* *p* < 0.05).

The control group was differentiated into adipocytes, and morphological alterations were observed due to the accumulation of lipid droplets in the cytoplasm. As evidenced by Oil Red O staining, The NAC and rutin treated groups had decreased lipid accumulation compared with the control. In addition, Figure 3 shows that TBWE treated group had suppressed lipid accumulations of approximately 28.8% compared with the control. In addition, TBWE group was slightly decreased adiponectin mRNA in 3T3-L1 cells as shown in Figure 4.

**Figure 5:**
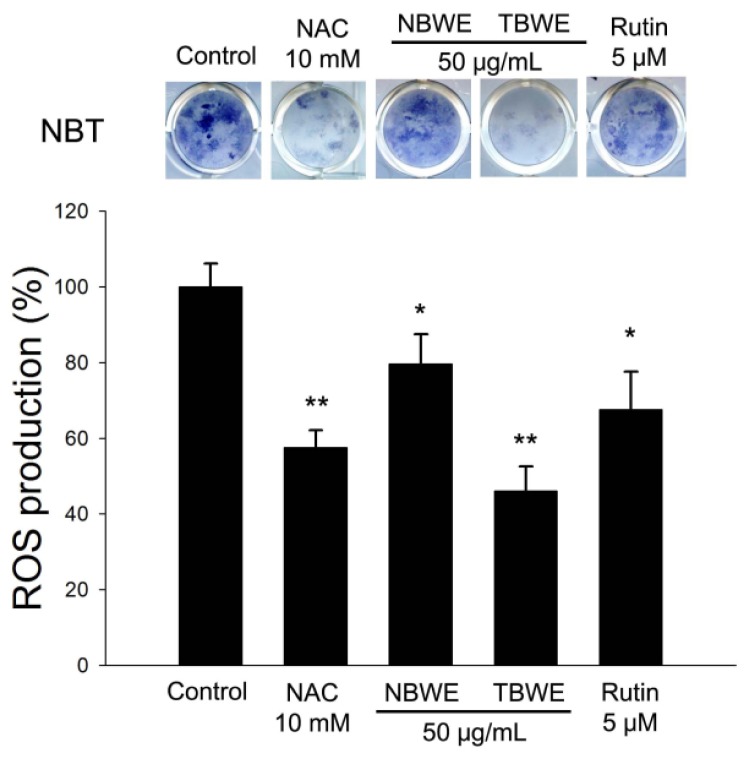
The effect of Buckwheat sprout extracts treated with MeJA (TBWE) on ROS production. Reduction of NBT to formazan by TBWE using the NBT assay. The results are given as the mean ± standard deviation of three replicates. Within each treatment group, the means without a common letter differ significantly (* *p* < 0.05; ** *p* < 0.01).

**Figure 6:**
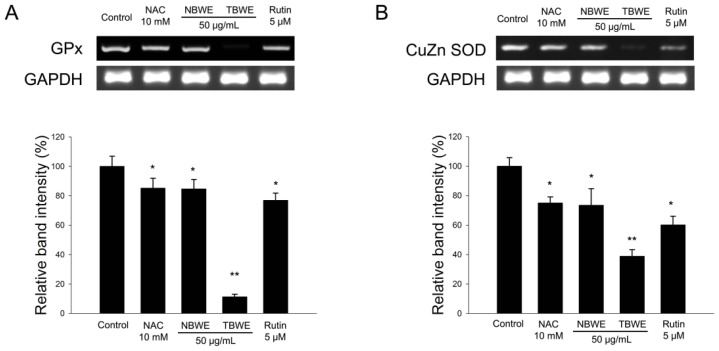
The effect of Buckwheat sprout extracts treated with MeJA (TBWE) on oxidative stress-related mRNA expression. The mRNA expression of (**A**) GPx and (**B**) Cu/Zn SOD were measured in 3T3-L1 cells. The results are given as the mean ± standard deviation of three replicates. Within each treatment group, the means without a common letter differ significantly (* *p* < 0.05; ** *p* < 0.01).

The NBT assay is a well-established technique used to quantify cellular oxidative metabolism [25]. Thus, we investigated the effect of TBWE on ROS production in adipocytes using the NBT assay. The levels of ROS production in fully differentiated 3T3-L1 cells significantly decreased following treatment with TBWE during adipogenesis compared with the control group (Figure 5), suggesting that TBWE has anti-oxidative effects on adipogenesis. It is also known that oxidative stress affects the expression of genes for GPx and Cu/Zn SOD. The GPx and Cu/Zn SOD were significantly lower in the TBWE treated group than in the control group (Figure 6).

**Figure 7:**
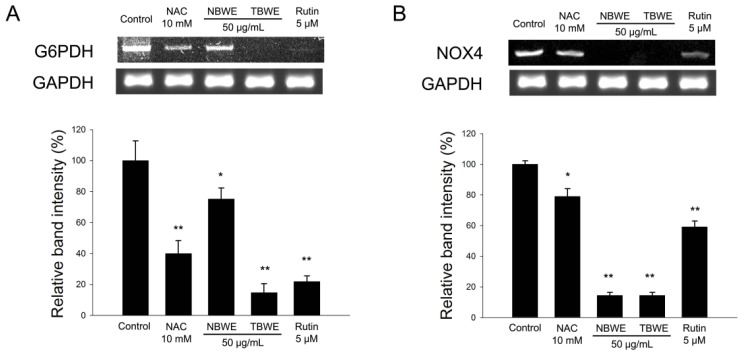
The effect of Buckwheat sprout extracts treated with MeJA (TBWE) on G6PDH and NOX4 mRNA expression. The mRNA expression of (**A**) G6PHD and (**B**) NOX4 were measured in 3T3-L1. The results are given as the mean ± standard deviation of three replicates. Within each treatment group, the means without a common letter differ significantly (* *p* < 0.05; ** *p* < 0.01).

Furthermore, TBWE dramatically suppressed the release of pro-oxidant enzymes such as NOX4 and the NADPH-producing G6PDH in Figure 7. Those results suggest that TBWE regulates lipid accumulation and ROS production; this finding implicates the GPx and Cu/Zn SOD enzymes during adipogenesis.

### 2.5. Discussion

The aim of this study was to determine the adipogenesis prevention potential of TBWE in 3T3-L1 adipocytes. 3T3-L1 preadipocytes are a useful cell line model for investigating adipogenesis. The differentiation of preadipocytes into adipocytes is accompanied by many changes in gene expression, such as a dramatic increase in the expression of aP2, followed by the expression of C/EBPα and PPARγ [26]. We investigated whether the effect of TBWE on differentiation and lipid accumulation in adipocytes is influenced by the adipocyte-related key transcription factors C/EBPα, PPARγ and their target genes, including aP2. In the present study, the control group showed increased adipocyte differentiation, which was associated with up-regulated expressions of C/EBPα, PPARγ and aP2 in differentiated 3T3-L1 adipocytes. Conversely, NBWE or rutin treated groups inhibited adipocyte differentiation and was accompanied by decreases in the expression of C/EBPα, PPARγ and aP2 during adipogenesis compared with the control group. These data are consistent with previous studies showing that rutin and NBWE can inhibit the adipocyte differentiation or reduce the occurrence rate of obesity [12,21]. Although it showed slight difference between Figures 2 and 3, these inconsistent potentially indicate that lipid accumulation is not only regulated by aP2 gene, but might be modulated by other feedback mechanism such as oxidative stress [27]. The TBWE treated group strongly suppressed adipocyte differentiation and was accompanied by a decrease in the expression of adipogenic markers during 3T3-L1 differentiation compared with both the rutin and NBWE treated groups. In addition, we found that the TBWE treated group had decreased lipid accumulation (Figure 3). These results indicate that the rutin content of TBWE and NBWE was 2.99 and 2.09 mg/g, respectively (Table 1). At a 50 μg/mL, TBWE contains the rutin content at a 0.15 μg/mL (0.25 μM) that it is lower concentration than rutin group (at 5 μM) used in this manuscript. Nevertheless, TBWE exhibited similar inhibition of lipid accumulation compared with rutin group (Figure 3). One of the reasons, TBWE contains various anti-adipogenic bioactive compounds such as rutin, chlorogenic acid, catechin, isoorientin, orientin *etc.* [18]. Combination of these bioactive compounds has synergistic activity compared to individual compounds. Lee and Lee [28] reported that although the phenolic compounds in crude extract including olive leaf extract showed strong *in vitro* activities to individual phenolics, antioxidant and antimicrobial activities of combined phenolics showed better than those of individual phenolics.

The levels of adipose tissue derived products such as interleukin-6, tumor necrosis factor-α and leptin are increased in obesity. On the other hands, adiponectin levels decrease in the obese [29]. Adiponectin acts as an autocrine and paracrine factor in adipose tissue by promoting of the gene expression responsible for adipogenesis *in vitro* [30]. Here, there were no difference of adiponectin mRNA expression among the control, NBWE and rutin. However, it is interestingly TBWE treated group decreased the expression of adiponectin mRNA compared to other groups. We have previously shown that TBWE significantly increased the phenolic compound amounts and the antioxidant activity [15]. According to our observations suggest that the inhibition of adipocyte differentiation has been enhanced mainly due to MeJA treatment on NBWE, which probably allows the improvement of biopharmaceutical activities, including anti-adipogenesis in TBWE.

The reduced production of ROS in the TBWE-treated groups caused a marked down regulation of the GPx and Cu/Zn SOD genes (Figures 5 and 6). Similar results have been previously reported in HepG2 cells [31] and support the hypothesis that TBWE regulates adipogenesis through the inhibition of ROS production, which induces the synthesis of the adipocyte markers, G6PDH and NOX4 (Figure 7). These data suggested the adiponectin and oxidative stress might have a close correlation [32]. Our results that show decreased ROS production caused by TBWE in adipocytes are of interest because similar evidence has implicated ROS signaling in atherosclerosis with obesity.

## 3. Experimental Section

### 3.1. Materials

Dulbecco’s modified Eagle’s medium (DMEM), bovine calf serum (BCS), fetal bovine serum (FBS), penicillin-streptomycin (P/S), phosphate-buffered saline (PBS), and trypsin-EDTA were purchased from Gibco (Gaithersburg, MD, USA). Dexamethasone (DEX), 3-isobutyl-1-methylxanthine (IBMX), insulin, Oil red O, NBT, and N-acetyl cysteine (NAC) were purchased from Sigma (St. Louis, MO, USA). All chemicals were also purchased from Sigma, unless noted otherwise.

### 3.2. Determination of Total Content of Phenolic Compounds

The total amount of phenolic compounds in the buckwheat sprouts was determined using Folin-Ciocalteu’s methods [18]. The methanolic extract (50 μL) was mixed with 450 μL of distilled water and 250 μL of 2 N Folin-Ciocalteu’s reagent. The mixture was then added to 1.25 mL of 20% Na_2_CO_3_. The resultant solution was incubated at 25 °C for 20 min and then centrifuged at 5000 rpm for 10 min. The absorbance of the supernatant solution was measured at 735 nm. A standard curve was prepared using gallic acid (GA), and the absorbance was converted to phenolic content in terms of milligrams of GA equivalent (GAE) per gram of dry weight (DW).

### 3.3. The Ferric Reducing Power (FRAP) Assay

The determination of the total antioxidant activity (FRAP assay) in the extract is a modified method of Benzie and Strain [33]. The stock solutions included 300 mM acetate buffer (3.1 g C_2_H_3_NaO_2_·3H_2_O and 16 mL C_2_H_4_O_2_), pH 3.6, 10 mM TPTZ (2,4,6-tripyridyl-*s*-triazine) solution in 40 mM HCl, and 20 mM FeCl_3_·6H_2_O solution. The fresh working solution was prepared by mixing 25 mL acetate buffer, 2.5 mL TPTZ, and 2.5 mL FeCl_3_·6H_2_O. The temperature of the solution was raised to 37 °C before use. Plant extracts (150 μL) were allowed to react with 2850 μL of the FRAP solution for 30 min in the dark condition. Readings of the colored product (ferrous tripyridyltriazine complex) were taken at 593 nm. The standard curve was linear between 200 and 1000 μM FeSO_4_.

### 3.4. The Oxygen Radical Absorbance Capacity (ORAC) Assay

Antioxidant capacity was determined by oxygen radical absorbance capacity (ORAC) assay following procedures previously described by [34]. This assay measures the ability of antioxidant components in test materials to inhibit the decline in R-PE fluorescence that is induced by a peroxyl radical generator, AAPH. The reaction mixture contained 1.7 mL of 75 mM phosphate buffer (pH 7.0), 100 μL of R-PE (3.4 mg/L), 100 μL of 320 mM AAPH, and 100 μL of sample. Phosphate buffer was used as a blank and Trolox (a water-soluble α-tocopherol analogue) as a standard during each run. The final volume of 2 mL was used in a 10 mm wide fluorometer cuvette. R-PE, phosphate buffer, and samples were pre-incubated at 37 °C for 15 min. The reaction was started by addition of AAPH. Fluorescence was measured and recorded every 5 min at emission of 570 nm and excitation of 540 nm using a Sequoia-Turner Model 450 fluorometer (Englewood, NJ, USA) until the fluorescence of the last reading declined to <5% of the first reading. One blank, one standard, and a maximum of 10 samples were analyzed at the same time. Each sample was repeated at least three times. The ORAC value refers to the net protection area under the quenching curve of R-PE in the presence of an antioxidant. The final results (ORAC value) were calculated and expressed using Trolox equivalents per gram of fresh-frozen weight.

### 3.5. Cultivation and Extraction of Buckwheat Sprout Treated with 0.1 mM MeJA

Buckwheat sprout treated with 0.1 mM MeJA was prepared as described previously [18]. Buckwheat seeds (40 g) were washed and soaked in distilled water at 25 °C for 4 h, and then the seeds were placed in a tray (32 × 6 × 2.8 cm) with cheese cloth. Four separated trays were placed in a commercial sprout cultivator with an autospraying system. The sprouts were cultivated in the dark at 18 °C (2 °C) for 7 d, with water automatically sprayed for 30 min every 12 h. For the application of MeJA to the sprouts, 0.1 mM MeJA (100 mL) dissolved in 0.25% ethanol was sprayed each day on the sprouts. As a control, 0.25% ethanol was sprayed on a different set of sprouts. No difference between samples treated with ethanol and water was observed. The sprouts were harvested at different cultivation times (0, 1, 3, 5, and 7 d). Samples on day 0 were the sprouts before MeJA treatment. Some of the harvested sprouts were immediately lyophilized for antioxidant measurements, while others were stored at −70 °C for enzymatic assays.

A ground buckwheat sprout sample treated with MeJA (0.1 g) was mixed with 2 mL of 80% methanol, and the mixture was shaken at room temperature for 12 h. After centrifugation at 12,000 rpm for 10 min, the supernatant solution was then filtered through a Whatman filter paper (No. 2), concentrated with a vacuum evaporator, and completely dried with a freeze drier. We also used same process to prepare control sample.

### 3.6. Cell Culture

3T3-L1 preadipocytes obtained from the American Type Culture Collection (ATCC, CL-173) were cultured, maintained, and differentiated as described by Lee *et al.* [6]. In brief, cells were plated and grown in DMEM with 3.7 g/L sodium bicarbonate, 1% P/S and 10% BCS. Adipocyte differentiation was induced via 2 days of treatment with post-confluent cells with 10% FBS and a hormonal mixture (MDI) consisting of 0.5 mM IBMX, 1.0 μM DEX, and 1.67 μM insulin. Two days after the initiation of differentiation, the culture medium was replaced with DMEM supplemented only with 1.67 μM insulin and 10% FBS. This medium was then replenished every other day. For the treatments, 2 day post-confluent cells were differentiated with or without buckwheat sprout extracts (50 μg/mL), NAC (10 mM ≈ 1.63 mg/mL), rutin (5 μM).

### 3.7. Cell Viability Assay

Cell viability was detected by using the 2,3-Bis(2-methoxy-4-nitro-5-sulfophenyl)-2*H*-tetrazolium-5-carboxanilide inner salt (XTT) assay (WelGene, Seoul, Korea). When cells were cultured to the log phase, they were seeded on a 96-well plate (1 × 10^5^ cells/well) for 24 h. Cells were divided into a control group and the treatment groups at the concentrations indicated. Absorbance (A) was detected with an enzyme calibrator at 450 nm. Cell viability = (A of study group/A of control group) × 100%. There were six wells for each concentration.

### 3.8. Determination of Lipid Accumulation

The extent of differentiation reflected by the amount of lipid accumulation was determined on day 8 via Oil red O staining. In brief, the cells were fixed in 10% formaldehyde in PBS for 1 h, washed with distilled water, and dried completely. Cells were stained with 0.5% Oil red O solution in the ratio of 60:40 (*v*/*v*) isopropanol:H_2_O for 30 min at room temperature and washed four times in water, then dried. Differentiation was also monitored under a microscope and quantified via elution with isopropanol and optical density (OD) measurements at 490 nm [6].

### 3.9. ROS Production in 3T3-L1 Preadipocyte

3T3-L1 preadipocytes were grown to confluence and induced to differentiate into adipocytes, as described. ROS production was detected via a NBT assay. NBT was reduced by ROS to a dark-blue, insoluble form of NBT called formazan [35]. On day 8 after induction, the cells were incubated for 90 min in PBS containing 0.2% NBT. Formazan was dissolved in 50% acetic acid, and the absorbance was determined to be 570 nm.

### 3.10. RNA Extraction and Semi-Quantitative RT-PCR

Total RNA was extracted from mature 3T3-L1 adipocyte cells with TRIzol reagent (Invitrogen, Carlsbad, CA, USA) in accordance with the manufacturer’s recommended protocols. RNA samples with OD_260_/OD_280_ ratios higher than 2.0 were employed for semi-quantitative RT-PCR. One microgram of total RNA was employed for the production of cDNA, using a reverse transcription-polymerase chain reaction (RT-PCR) system. All the primers used in this study are listed in Table 2. The PCR products were then run on 1.5% (*v*/*v*) agarose gels, stained with ethidium bromide, and photographed. The expression levels were quantified via scanning with a gel documentation and analysis system (Image J Program, NIH, Bethesda, MD, USA).

### 3.11. Statistical Analysis

All experiments were repeated three times. The results were statistically analyzed via ANOVA and Duncan’s multiple range tests. A *p*-value of <0.05 was considered statistically significant.

## 4. Conclusions

We demonstrated that the transcription of C/EBPα and PPARγ dramatically decreases in the presence of TBWE, which is a better result than that found for plain rutin compound and NBWE, which in turn down-regulates or inactivates aP2. The reduction in active aP2 leads to the attenuation of NOX4 and G6PDH mRNA expression during adipogenesis in 3T3-L1 cells and the suppression of adipocyte differentiation [36]. Furthermore, TBWE also blocks the accumulation of lipids, ROS contents and adiponectin in adipocytes. Together, our results demonstrated that TBWE strongly increase the phenolic compound compared to NBWE and significantly suppresses adipogenesis through the inhibition of major adipocyte markers, and also NOX and ROS production, compared with NBWE or rutin. These findings strongly indicate that TBWE has potential as an adipogenesis prevention substance with antioxidant properties.

## Figures and Tables

**Table 1:** Total phenolic compound and antioxidants activities of buckwheat sprout extracts treated with MeJA (TBWE).

	Total phenolic compound (mg/g)	Rutin (mg/g)	FRAP Assay (mM Fe^2+^/g of dried sample)	ORAC Values (μM trolox)		

				Hydrophilic	Lipophilic	Total
NBWE	14.4 ± 0.4	2.09 ± 0.27	0.09 ± 0.01	60.6 ± 3.8	104.0 ± 5.1	164.6 ± 6.3
TBWE	22.9 ± 0.6 **	2.99 ± 0.11 **	0.21 ± 0.02 **	103.3 ± 19.2 *	102.5 ± 7.0	205.8 ± 25.8

* *p* < 0.05;

** *p* < 0.01 *vs.* NBWE.

**Table 2:** Primer sequences for semi-quantitative RT-PCR analysis.

Primers	Sequences	

	Forward	Reverse
PPARγ	CCAGAGTCTGCTGATCTGCG	GCCACCTCTTTGCTCTGATC
CEBPα	GGTGCGCAAGAGCCGAGATAAAG	AGTTCACGGCTCAGCTGTTCCAC
aP2	GACCTGGAAACTCGTCTCCA	CATGACACATTCCACCACCA
GPx	CTCGGTTTCCCGTGCAATCAG	GTGCAGCCAGTAATCACCAAG
Cu/Zn-SOD	CAGCATGGGTTCCACGTCCA	CACATTGGCCACACCGTCCT
Adiponectin	CATGACCAGGAAACCACGACT	TGAATGCTGAGCGGTAT
NOX4	GAAGCCCATTTGAGGAGTCA	GGGTCCACAGCAGAAAACTC
G6PDH	CGATGGCAGAGCAGGT	GATCTGGTCCTCACG
GAPDH	AACTTTGGCATTGTGGAAGG	ACACATTGGGGGTAGGAACA
